# Protective effects of enoxaparin treatment against uterus ischemia/reperfusion injury in rats

**DOI:** 10.1590/acb407225

**Published:** 2025-09-29

**Authors:** Duygu Lafci, Mustafa Hilmi Yaranoglu, Eren Altun, Eray Metin Guler, Ceyda Sancakli Usta

**Affiliations:** 1 Balikesir Univesity – School of Medicine – Department of Obstetrics and Gynecology – Balikesir – Turkey.; 2 Beylikduzu State Hospital – Department of Obstetrics and Gynecology – Istanbul – Turkey.; 3 Balikesir Univesity – Experimental Research Center – Balikesir – Turkey.; 4 Bagcilar Training and Research Hospital – Department of Pathology – Istanbul – Turkey.; 5 Haydarpasa Numune Health Application and Research Center – Clinic of Medical Biochemistry – Istanbul – Turkey.; 6 University of Health Sciences – Faculty of Hamidiye Medicine – Department of Medical Biochemistry – Istanbul – Turkey.

**Keywords:** Enoxaparin, Ischemia, Reperfusion Injury, Uterus, Oxidative Stress

## Abstract

**Purpose:**

This study analyzed the protective effects of enoxaparin in rat uteruses by exposing the uterus to experimental ischemia-reperfusion injury.

**Methods:**

Thirty female rats were homogenized by weight and cycle and divided into three groups: a control group, an ischemia-reperfusion (I/R) group, and an ischemia-reperfusion plus enoxaparin (I/R+E) group. An experimental uterine I/R model was established in the I/R and I/R+E groups. Unlike I/R group, all rats in the I/R+E group received subcutaneous enoxaparin at 0.5 mg/kg 2 hours before ischemia. In histopathological analysis, endometrial glandular and endo/myometrial stromal changes were scored according to the histopathological scoring system. Biochemically, catalase (CAT), superoxide dismutase (SOD) enzyme activities, and malondialdehyde (MDA) levels were measured in uterine tissues.

**Results:**

In histopathological analysis, all of the control group’s scores were lower than those of the other groups, except for necrosis (*p* 0.05). There was no significant improvement in the glandular and stromal changes between I/R and I/R+E (*p* 0.05). However, the I/R+E group showed significantly increased SOD and CAT activities and decreased MDA levels compared to the I/R group (*p* = 0.000).

**Conclusion:**

Although enoxaparin did not significantly improve histopathological injury, its potent antioxidant effects suggest a protective role against oxidative stress in uterine I/R injury.

## Introduction

Uterine transplantation (UTx) has become the ideal treatment method for having a child and provides women with uterine-induced infertility a means to carry their genetic offspring without the legal complications of surrogacy^1^. It has been reported that absolute uterine factor infertility, which affects one in every 500 women in the reproductive period of their lives, is caused by uterine defect or absence^2^. The uterine transplant operation, the first case of which was reported in 2000 and ended in the removal of the uterus three months due to graft necrosis, is a form of treatment that continues to make significant progress nowadays, including a process that led to the realization of the first human birth after UTx by the Brännström team in 2014^1 ^–^ 3^. Only a small number of successful UTx cases have been reported, and most of them have been achieved through living donors.

The success of UTx largely depends on the ability to minimize ischemia-reperfusion (I/R) injury^1^. Reactive oxygen species, cell damage, apoptotic processes, inflammation, necrosis, and microvascularization disorders, which play key roles in ischemia and reperfusion, can significantly impair overall transplant success. Therefore, investigating the preventive effects of pharmacological agents on this damage is important for clinical success^1^^–^ 3.

Various pharmacological agents, such as dexmedetomidine, remifentanil, mycophenolate mofetil, relaxin, erythropoietin, melatonin, glycine, crocetin, and resveratrol, have been studied to analyze their ability to prevent or reduce oxidative stress and I/R damage that may occur in uterine I/R models^4^^,^ 5. However, no study has been found in the literature that shows the effect of enoxaparin on uterine ischemia in rat models.

Low-molecular-weight heparins (LMWH) are glycosaminoglycans used as anticoagulants in both thrombosis prophylaxis and the treatment of acute thrombotic events; they cause a decrease in reactive oxygen species and are known to have anti-inflammatory effects. Enoxaparin was the drug selected for our study, because it is frequently used in the field of gynecology and obstetrics for the prevention of thromboembolism, and it is thought that its known anticoagulant, antioxidant, and anti-inflammatory effects will be helpful in the successful completion of UTx in the future^6^^,^ 7. This study may serve as a foundation to improve the success rates of uterine transplantation.

## Methods

This study was conducted after approval by the Balikesir University Animal Experiments Local Ethics Committee, with the number 2024/10-4 dated October 24, 2024, and within the framework of the rules outlined in the Balikesir University Animal Experiments Local Ethics Committee Directive.

**Figure 1:**
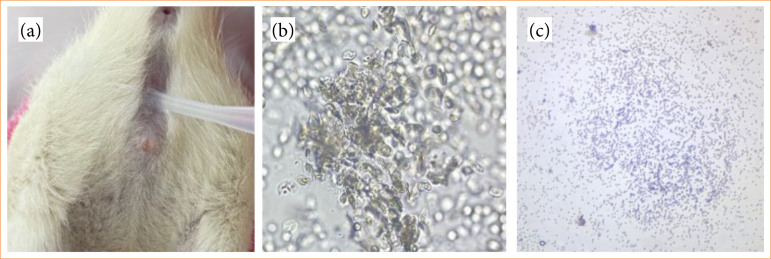
Vaginal smear. **(a)** Vaginal smear collection method. **(b)** Estrous phase in light microscopy x50, cornified squamous epithelial cells giving the appearance of leaves scattered in clusters. **(c)** Diestrus phase in which the amount of nucleated cells increases, light microscopy x50.

Source: Elaborated by the authors.

Thirty Wistar albino female rats weighing between 210–250 g were used for the study. Power analysis performed to calculate the minimum sample size required for the study (alpha error = 0.05; 1-beta = 0.8) showed that ≥ nine rats were required for each study group^6^. All rats were housed in a controlled environment with constant temperature and cycle (22 ± 1 °C; 12 h/12 h light/dark cycle) with free access to food and water. Rats with at least three estrous cycles were included in the study. The cycle was performed according to the vaginal smear collection method shown by Marcondes et al.^8^. In order to ensure homogenization between the groups, the female rats in the study were included in the diestrus phase (Fig. 1)^8^. The experimental I/R model was performed using the previously applied and validated model shown in the literature^5^. The groups were divided into three groups with 10 rats in each group by random distribution of rats:

- Group 1: control group (*n* = 10);
- Group 2: I/R group (*n* = 10);
- Group 3: I/R and enoxaparin group (I/R+E; *n* = 10).

All rats were anesthetized with 80 mg/kg ketamine hydrochloride (Ketasol; Richter Pharma) and 10 mg/kg xylazine hydrochloride (Rompun; Bayer Health Care). Repeated intramuscular administration of ketamine hydrochloride (50 mg/kg) and xylazine hydrochloride (10 mg/kg) was used as needed to maintain anesthesia. After anesthesia was achieved, the abdominal area of the rats placed in the supine position was shaved, and 10% povidone-iodine was used for disinfection. Laparotomy was performed with a 2–3-cm vertical incision in the midline of the lower abdomen. For group 1, the uterus was exposed by pulling the intestines aside, and both uterine horns were removed. For group 2, the abdominal aorta was exposed, and uterine I/R was created by applying an atraumatic microvascular clamp (Bulldog clamp; Aesculap, B. Braun Melsungen) to the distal abdominal aorta. The clamp was placed just above the bifurcation of the iliac arteries and vascular pedicle.

**Figure 2:**
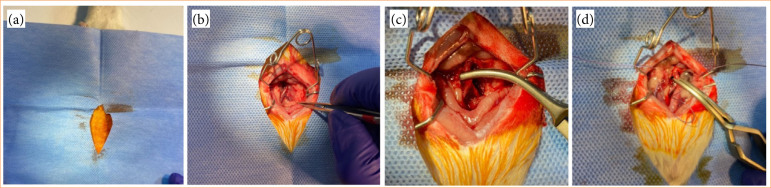
Surgical procedure. **(a)** Surgical preparation. **(b)** Exposure of the abdominal aorta. **(c)** Clamping of the distal abdominal aorta with Buldogg clamp. **(d)** Ligating bilateral ovaries with Vicrl suture.

Source: Elaborated by the authors.

To prevent collateral circulation, bilateral ovaries were ligated with a polyglycolic acid suture (Vicryl; Johnson and Johnson Medical, Ethicon) (Fig. 2). The incision line was covered with moist gauze to minimize heat and fluid loss in rats subjected to 1 hour of ischemia. In I/R groups, a 1-hour ischemia period was followed by 1-hour reperfusion. In group 3 (I/R+E group), ischemia was performed as in the I/R group; however, subcutaneous Enoxaparin (Clexane 4,000 anti-Xa/0.4 mL solution for injection ready-to-use injector, Sanofi) 0.5 mg/kg was administered to all rats in this group 2 hours before starting these procedures. The dose of enoxaparin given 2 hours before inducing ischemia was determined to be similar to the 40-mg/day dose determined for thromboembolism prophylaxis in humans in previous studies^9^. In groups 2 and 3, both uterine horns were removed immediately after reperfusion. For all groups, the right uterine horn was stored in the freezer at -80°C for histopathological examination and the left for biochemical analysis. Rats were sacrificed using the exsanguination method during surgery.

### Histologic evaluation

**Figure 3:**
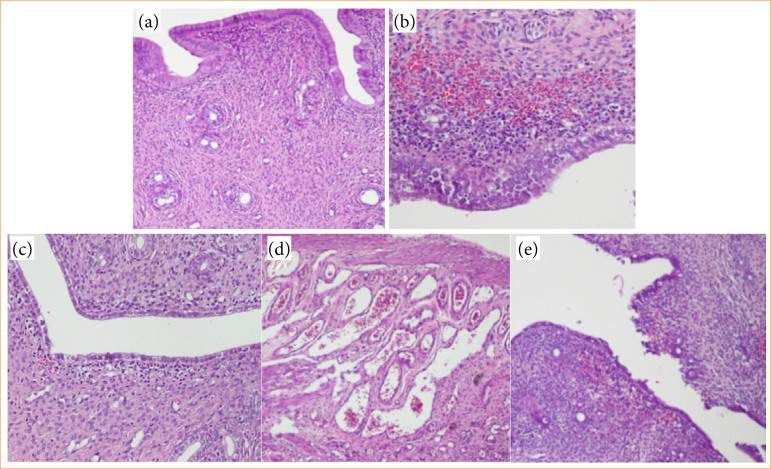
Histopathological morphological changes. **(a)** Control group; normal endometrial glandular structures and stroma. **(b)** Significant inflammation and hemorrhage under the epithelium in the ischemia/reperfusion (I/R) group. **(c)** Partial inflammation and hemorrhage under the epithelium in the enoxaparin group (I/R+E). **(d)** Significant congestion and edema in the myometrial layer in the I/R group. **(e)** Focal areas of shedding, degeneration, and hemorrhage in the endometrial surface epithelium in the enoxaparin group (I/R+E). (a–e) Hematoxylin and eosin, original magnification x50, x100, x200.

Source: Elaborated by the authors.

For histopathological analysis, the uterus was divided into anterior and posterior parts on the vertical axis to see the endometrial cavity. The pieces were placed in a 10% buffered formalin solution and kept in a fixation solution for 24 hours. Paraffin blocks were obtained from the tissues processed through alcohol xylene and paraffin stages within the scope of routine procedures. Five-µm thick consecutive thin slices were cut from the uterine tissues embedded in paraffin blocks with a microtome (Leica RM2135, Wetzlar, Germany) and placed on a slide. Hematoxylin and eosin (H&E) staining was performed on the obtained uterine tissue sections from the study groups, which were analyzed with a light photomicroscope (Olympus BX; Olympus Co, Tokyo, Japan) using 10x and 20x objectives and photographed (Fig. 3).

Histopathological analyses were performed using a scoring system based on morphological changes and revised according to the relevant literature^5^^,^ 10 ^,^ 11. The scoring included:

- endometrial epithelial glandular changes (inflammatory cell infiltration, glandular cell degeneration);
- endo/myometrial stromal changes (congestion and hemorrhage, necrosis, edema, vascular thrombosis).

Each histopathological finding was scored between 0 and 2 based on the severity of I/R injury, as detailed in Table 1.

**Table 1:** Histopathological morphological changes and scoring system.

Histopathological morphological changes		Score		
		0	1	2
**Endometrial epithelial glandular changes**	Inflammatory cell infiltration	None	Moderate infiltration	Severe infiltration
	Endometrial glandular cell degeneration	None	< 20%	> 20٪
**Endo/myometrial stromal changes**	Congestion and hemorrhage	None	Subendometrial	Myometrial and endometrial
	Necrosis	None	< 20%	> 20%
	Edema	None	< 50٪	> 50%
	Vascular thrombosis	None	In < 50% of vascular structures	In > 50٪ of vascular structures

Source: Elaborated by the authors.

### Biochemical evaluation

Following the weighing of uterine samples, tissues were homogenized in 0.1-mol/L phosphate-buffered saline (PBS, pH 7.4) at a 1:9 (w/v) ratio using ceramic beads in a QIAGEN TissueLyser LT homogenizer (Hilden, Germany) for 10 minutes. The homogenates were then centrifuged at 10,000 × g for 10 minutes at +4°C using a Beckman Coulter Allegra X-30 centrifuge (IN, United States of America), and the supernatant and pellet were separated^12^.

### Total protein measurement

The total protein content in the supernatants was quantified using a commercial Bradford-based assay kit (Coomassie Plus Protein Assay, Thermo Fisher Scientific, Massachusetts, United States of America). The Bradford assay is based on the binding of Coomassie brilliant blue dye to proteins under acidic conditions, resulting in a color change from brown to blue^13^.

Five μL of 1:10 diluted uterine tissue supernatant (with 1× Dulbecco’s PBS) were added to microplate wells, followed by 200 μL of Bradford reagent. After a 5-minute incubation at room temperature, absorbance was measured at 595 nm using a spectrophotometer (BioTek Synergy HTX Multimode Reader). Total protein levels were calculated based on a standard curve.

### Measurement of catalase, superoxide dismutase, and malondialdehyde levels

The levels of superoxide dismutase (SOD, BTLab, E0168Ra), catalase (CAT, BTLab, E0869Ra), and malondialdehyde (MDA, BTLab, E0156Ra) in the supernatants were determined using commercial enzyme linked immuno sorbent assay (ELISA) kits according to the manufacturer’s instructions.

For each parameter (SOD, CAT, MDA), 40 μL of sample and 10 μL of the specific antibody were added to the microplate wells, followed by 50 μL of streptavidin-HRP. The plates were incubated at 37°C for 60 minutes. After washing the wells five times with 300 μL of wash buffer, 50 μL each of substrate solutions A and B were added. The plates were incubated in the dark at 37°C for 10 minutes. The reaction was stopped by adding 50 μL of stop solution, and absorbance was measured at 450 nm. SOD and CAT concentrations were expressed as ng/mL/mg protein, while MDA levels were expressed as nmol/mL/mg protein^14^.

### Statistical analysis

The findings obtained from the study groups were examined with descriptive statistics. Analyses were evaluated using Statistical Package for the Social Sciences (SPSS) 23.0 (IBM Corp., Armonk, NY, United States of America). Experimental groups were analyzed using one-way analysis of variance (ANOVA) with Tukey’s honestly significant difference (HSD) or non-parametric Kruskal-Wallis tests. *p* < 0.05 was considered significant.

## Results

Since no exits occurred during the experiment, the experiment was finalized with the initial number of 30 rats.

In H&E-stained uterine tissue sections, there was no significant difference between the I/R+E group and the I/R group in terms of statistical analysis in all parameters. However, all endo/stromal glandular changes except necrosis had lower scores in the I/R+E group. When I/R groups were compared separately with the control group, all histopathological changes except necrosis and vascular thrombosis were statistically significant and higher in I/R groups (*p* < 0.05). Necrosis formation was observed in only one rat in the control group.

The total score of all changes was calculated as 14 in the control group, 70 in the I/R group, and 64 in the I/R+E group. While the control group was statistically significantly lower than the I/R groups (*p* < 0.05), there was no statistically significant difference between the I/R and I/R+E groups (*p* > 0.05) (Table 2).

**Table 2:** Comparison of histopathological scores of the groups (one way analysis of variance; differences between groups were analyzed using post-hoc Tukey’s honestly significant difference).

	Group	n	Mean ± SD	Min–Max	Post-hoc *p* -value
**Endometrial epithelial glandular changes**	1	10	0.40 ± 0.51	0–1	1–2: < 0.05^x^ 1–3: < 0.05^x^ 2–3: > 0.05
	2	10	2.8 ± 1.13	1–4	
	3	10	2.8 ± 0.78	2–4	
**Endo/myometrial stromal changes**	1	10	1.0 ± 1.63	0–4	1–2: < 0.05^x^ 1–3: < 0.05^x^ 2–3: > 0.05
	2	10	4.2 ± 1.03	2–6	
	3	10	3.6 ± 1.34	2–6	
**Total score**	1	10	1.4 ± 1.83	0–4	1–2: < 0.05^x^ 1–3: < 0.05^x^ 2–3: > 0.05
	2	10	7.0 ± 1.82	4–10	
	3	10	6.4 ± 2.01	4–10	

*Statistically significant (*p* < 0.05).

Source: Elaborated by the authors.

**Figure 4:**
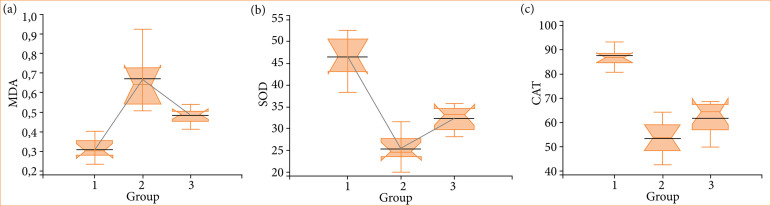
Enoxaparin’s effect on malondialdehyde (MDA), superoxide dismutase (SOD), and catalase (CAT) levels. **(a)** Effect of enoxaparin on malondialdehyde levels. **(b)** Effect of enoxaparin on superoxide dismutase activity. **(c)** Effect of enoxaparin on catalase activity. One way analysis of variance-Kruskal-Wallis test.

Source: Elaborated by the authors.

MDA levels were found to be significantly elevated in the I/R group compared to the control group (*p* = 0.000). When the I/R group was compared with the I/R+E group, enoxaparin administration significantly decreased MDA levels (*p* = 0.000). Furthermore, a decline in SOD activity was observed in the I/R group in comparison to the control group (*p* = 0.000). However, enoxaparin administration significantly increased SOD activity in the I/R+E group compared to the I/R group (*p* = 0.000). A similar outcome was observed in CAT activity, which also demonstrated the same result as SOD activity between the groups. Furthermore, enoxaparin significantly increased SOD activity in comparison to the I/R group (*p* = 0.000) (Fig. 4).

## Discussion

It has been stated that approximately one million women do not have a chance of pregnancy other than transplantation for various reasons^5^^,^ 10 ^,^ 15 ^,^ 16. However, it has been observed that I/R injury occurring during transplantation is closely related to acute and chronic graft rejection^15^. Prevention of reactive oxygen species-mediated I/R is a successful therapeutic strategy in transplantation models^17^. The protective effect of enoxaparin against uterine I/R injury was evaluated in the study. Although the results did not show statistically significant results regarding histopathological damage levels between the I/R+E and I/R groups, less damage was observed in the enoxaparin group. Notably, it demonstrates that enoxaparin reduces oxidative stress (as indicated by MDA levels) and enhances the activities of antioxidant enzymes CAT and SOD.

In this study, we observed that enoxaparin has beneficial effects in protecting against uterine I/R damage. Studies investigating the protective effect against I/R injury in the literature aim to benefit from the antioxidant and anti-inflammatory effects of the drugs. Enoxaparin, a LMWH known for its anticoagulant effect, has also been reported to exhibit anti-inflammatory and antioxidant activity. It has been studied to investigate its protective effect against I/R injury in various tissues, including ovarian, cerebral, hepatic, lower extremity, and cardiac tissues^18^^–^ 22. These studies emphasized that LMWHs help prevent cellular damage by reducing inflammatory markers, such as MDA, and lipid peroxidase levels.

The I/R injury resulting from reperfusion is a complex situation^11^^,^ 23. The return of oxygen to the tissue with reperfusion after ischemia, in which anaerobic metabolism is dominant, and calcium levels increase, creates chemical reactions in which xanthine oxidase converts excess hypoxanthine into reactive oxygen radicals. Antioxidants, defense mechanisms against rapidly increasing levels of these radicals, are insufficient^4^^,^ 11 ^,^ 23. The study examined antioxidant mechanisms including SOD and CAT and found that enoxaparin increased the activity of these antioxidants. In addition, high amounts of reactive oxygen radicals cause lipid peroxidation as indicated by MDA, which increases damage by increasing membrane permeability and intracellular ruptures^24^^–^ 26. Our study has shown that enoxaparin reduces MDA levels. LMWHs, which are widely known for their use in venous thromboembolism prophylaxis, are also frequently used drugs in the field of obstetrics to prevent pregnancy loss^6^^,^ 27.

To the best of our knowledge, this is the first study to investigate the protective effect of enoxaparin against uterine I/R damage. A study conducted on a rat model investigated the protective effect of enoxaparin against ovarian I/R injury and its effect on *in-vitro* fertilization results. Enoxaparin minimizes ovarian damage and protects ovarian reserve^22^. Another study was conducted on ovarian tissue, and an I/R model was created to determine tissue damage scores according to vascular congestion, hemorrhage, inflammatory cell infiltration, and cell degeneration parameters. Similar to this study, no significant difference was observed in the protective effect of enoxaparin on inflammatory cell infiltration and degeneration. The study, which detected significantly higher hemorrhage in the enoxaparin group, stated that this situation may be related to increased blood flow. However, it concluded that enoxaparin has a positive effect on ovarian reserve^6^.

In a study in which acute lung injury was induced in rats by cecal ligation and puncture (CLP), it was noted that similarly significant histopathological changes were not observed in the control group. However, all tissue change scores were lower in the LMWH+CLP group compared to the CLP group by itself^28^. A rat model study on renal transplantation demonstrated that LMWHs significantly reduced inflammation^29^. It has been reported that LMWHs also cause a decrease in inflammatory markers and oxidative stress concentrations in hemodialysis patients^30^.

One of the limitations of our study is that the 1-hour ischemia-1-hour reperfusion model we used does not reflect a clinical situation. Another limitation is that the vascular thrombosis score was lower in the I/R+E group compared to the I/R group, suggesting that enoxaparin was at therapeutic levels in the uterine tissue. However, the dosing schedule for enoxaparin was not applied^31^. Therefore, more research is needed on I/R injury as an experimental model.

## Conclusion

To our knowledge, this is the first study investigating the protective effect of enoxaparin during UTx surgery. The study demonstrated that enoxaparin was particularly effective in mitigating oxidative stress, likely due to its antioxidant properties. Further studies with larger schemes are needed to guide uterine UTx applications, which are planned to become increasingly widespread.

## Data Availability

The datasets used and/or analyzed during the current study are available from the corresponding author on reasonable request.
